# Between help and hindrance: a qualitative study on inclusion of birth companions in closed, invited and contested spaces within maternity care settings in Malawi

**DOI:** 10.1136/bmjph-2025-003706

**Published:** 2026-07-10

**Authors:** Bianca Kandeya, Wanangwa Chimwaza, Claudia Hanson, Dorcus Kiwanuka Henriksson, William Stones, Effie Chipeta, Helle Mølsted Alvesson

**Affiliations:** 1 Centre for Reproductive Health, Kamuzu University of Health Sciences, Blantyre, Southern Region, Malawi; 2 Department of Global Public Health, Karolinska Institute, Stockholm, Stockholm County, Sweden; 3 School of Global Public Health, Kamuzu University of Health Sciences, Blantyre, Malawi

**Keywords:** ethics, Attitude, Communication

## Abstract

**Background:**

Birth companionship is widely recognised as a key component of respectful maternity care, with well-documented benefits for maternal and neonatal outcomes. However, in low-resource settings like Malawi, its integration into facility-based care remains limited and uneven due to systemic, infrastructural and interpersonal challenges. The study aimed to explore how birth companionship is negotiated in closed, invited and contested spaces within maternity care settings.

**Methods:**

This qualitative exploratory study, nested within the ALERT (Action Leveraging Evidence to Reduce Perinatal Mortality and Morbidity in sub-Saharan Africa) project, was conducted in one government and one faith-based hospital in Malawi. 65 Participants were purposively sampled: 24 postpartum women, 20 birth companions and 21 maternity care providers. Data were collected through semi-structured interviews, focused group discussions with providers and birth companions, 7-day provider diaries and 72 hours of observations involving 16 birthing mothers and 16 maternity care providers. Transcripts were uploaded into NVivo V.14 for coding and data were analysed using reflexive thematic analysis, applying Gaventa’s Spaces of Power framework for interpretation.

**Results:**

Findings show that companion inclusion was overlooked in maternity ward design and policy, leaving involvement unstructured and dependent on provider discretion. Provider-centred routines, lack of guidance and training, overcrowding, restricted visiting hours and inadequate facilities reinforced closed spaces and limited continuous support. In invited spaces, companions offered emotional and practical support, but participation remained conditional, poorly acknowledged and shaped by provider needs. In contested spaces, unclear roles generated tension over authority and responsibility as companions sought visibility, questioned decisions or mediated between women and providers, often risking reprimand.

**Conclusion:**

The integration of birth companionship in Malawi’s maternity care system remains conditional and inconsistently practised. Addressing this requires clear policy frameworks, role orientation for companions, infrastructural investment and provider training. Interventions should promote well defined inclusive spaces where companions are empowered and midwives supported, thereby advancing respectful, woman-centred care.

**WHAT IS ALREADY KNOWN ON THIS TOPIC**

- Birth companionship is recognised globally as a key component of respectful maternity care, with proven benefits for maternal and newborn outcomes. However, birth companionship remains inconsistently implemented in low-resource settings like Malawi, where systemic, infrastructural and sociocultural barriers, as well as limited understanding of real-time power dynamics, hinder its integration.

**WHAT THIS STUDY ADDS**

- This study reveals how power dynamics, shaped by provider discretion, policy gaps and structural constraints, influence the inclusion of birth companions in Malawi’s maternity care, and how they unfold within closed, invited and contested spaces.
- It also shows how institutional rules, ward routines and provider discretion shape whether companions are excluded, tolerated or informally engaged in care.

**HOW THIS STUDY MIGHT AFFECT RESEARCH, PRACTICE OR POLICY**

- The findings highlight the need for clear policies, infrastructure and provider training to create inclusive, structured spaces for birth companions, offering a framework to guide woman-centred care and health system reform in low-resource settings.

## Introduction and background

 Provision of high-quality maternity care requires responsiveness to women’s needs, including the recognition of birth companions as a vital source of emotional, physical and advocacy support.^1^,^3^ Birth companions have been shown to reduce stress, provide comfort and improve communication between women and healthcare providers during labour.^4^ The WHO recommends allowing women to choose a companion of their choice, highlighting that continuous support during childbirth contributes to better maternal and neonatal outcomes, including shorter labour, improved Apgar scores and fewer caesarean deliveries.^4^,^8^ Companionship also promotes key principles of respectful maternity care by improving communication between providers and labouring mothers as well as enhancing women’s sense of agency and dignity during childbirth.^9 10^

Despite these benefits, there remains limited understanding of how companionship is experienced and negotiated in real-time within health facilities, particularly in low-resource settings.^2^ In such contexts, companions can be perceived variably, as helpful allies or disruptive intruders, especially when roles are unclear or when they challenge existing norms around privacy, authority or decision-making. These relational tensions can shape the quality of care received, especially in environments already strained by staffing shortages, limited infrastructure and overburdened health workers.^11^ Understanding how these interpersonal dynamics unfold in the labour ward is essential to improving respectful, woman-centred care.

Although global evidence supports the value of companionship, its integration into formal maternity care systems in resource-limited settings like Malawi remains uneven. Maternity services in Malawi are typically driven by provider-centred models that prioritise institutional efficiency over women’s preferences.^12 13^ Structural challenges, such as limited space, staff shortages and overcrowding, often restrict the presence and active participation of companions.^14 15^ Additionally, deeply rooted sociocultural norms surrounding modesty, shame and gender roles can discourage women from using companions, particularly in close-knit or traditional communities.^16^,^19^

Research from similar contexts indicates that privacy concerns, fear of judgement and cultural taboos can all serve as barriers to companionship. For example, women in Ethiopia, Kenya and Zambia have declined companionship due to fear of gossip or discomfort with having a support person present. These findings point to the importance not only of policy changes, but also of a shift in how maternity care is structured—moving toward models that acknowledge and support women’s choices and rights during childbirth.^15 20 21^

In Malawi, companionship has cultural roots, with female relatives or traditional birth attendants historically providing support during labour.^22 23^ This informal practice has continued into formal healthcare settings, where birth companions, often family members, play supportive roles.^24^ While these companions can improve outcomes by providing practical help and emotional reassurance, their integration into facility-based care remains minimal.^25^ Recent policy developments, such as the inclusion of birth companionship in Malawi’s 2021–2025 Sexual and Reproductive Health and Rights Policy, signal growing recognition of the practice, but meaningful implementation is still lacking.^25 26^

This study explores how birth companionship is shaped by power dynamics within Malawi’s maternity care settings. Specifically, it explores how power operates within what Gaventa describes as ‘closed’, ‘invited’ and ‘contested’ spaces, each representing different levels of access, participation and control.^27^ Closed spaces are controlled by dominant decision-makers and exclude external actors from participation. Invited spaces permit limited involvement of external stakeholders, but only under predefined terms set by those in authority. In contrast, contested spaces arise when less powerful groups actively challenge these constraints and assert their influence beyond formal institutional structures.

Power in health systems is not always visible or top-down; it also exists in hidden and invisible forms.^28^ Hidden power shapes agendas and determines who gets to participate in decision-making, while invisible power influences people’s beliefs and behaviours, often without their awareness. Research shows that power in health systems is shaped not only by institutional authority and donor influence but also by political, social and cultural dynamics, operating in both formal structures and everyday practices.^29^,^32^ Within maternity care, healthcare providers typically hold professional authority, while labouring women and their companions, especially in hierarchical or resource-poor settings, have limited capacity to express preferences or challenge norms. These imbalances deeply affect whether women feel respected, supported or silenced during childbirth.^30 33^

By applying the *Spaces of Power* framework, this study helps unpack how different forms of power influence inclusion or exclusion of companions during childbirth, with significant implications for women’s experiences. Shared power fosters respect and empowerment, while uncontrolled power can lead to marginalisation or neglect.^34^

## Methods

### Design

A qualitative exploratory study design was used to collect and analyse data. The project was nested within the Action Leveraging Evidence to Reduce Perinatal Mortality and Morbidity in sub-Saharan Africa (ALERT) project, which was supported by the European Union’s Horizon 2020 funding.^35^ The ALERT project aimed to evaluate a comprehensive intervention targeting intrapartum care, which included labour companionship. This study specifically draws on data collected during the initial co-design phase of the ALERT project.

### Study setting

The study was conducted in two high-volume maternity units in Malawi’s Central Region, one faith-based hospital and one government-run facility. These sites were purposively selected for high birth volumes and their representation of different service models, workloads and infrastructural conditions for implementing birth companionship. In Malawi, most hospitals are government-run (60%), while faith-based institutions, largely under the Christian Health Association of Malawi, and private facilities provide the remaining services, often in rural and underserved areas.

The faith-based hospital operates on a fee-for-service basis and recorded approximately 3000 births in 2021, supported by 40 midwives. By contrast, the government hospital provides free maternity care and recorded nearly 6000 births, with the same number of midwives.^36^ This illustrates the higher patient load commonly experienced in public facilities and the associated pressure on human resources.

The government facility operates a two-shift system, with about eight midwives per shift, managing approximately 30 deliveries per day. The faith-based facility uses a three-shift system, with around four midwives per shift, managing about 15 deliveries daily. Both units provide primary and secondary maternity care, but their different staffing patterns, workloads and organisational arrangements shape how companionship is implemented.

Both facilities promote birth companionship as part of respectful maternity care, consistent with national and global recommendations. However, Malawi’s high fertility rate, averaging 3.7 children per woman, places substantial pressure on maternity services, especially in public facilities facing overcrowding and limited resources.^37^ The faith-based hospital has infrastructure more supportive of continuous companionship, including private birthing rooms that allow companions to remain with women during labour and birth. In contrast, the public hospital serves a larger population under more constrained conditions and operates a shared labour ward with limited privacy.

### Researcher characteristics and reflexivity

Our research team included clinical and non-clinical members, including midwives, obstetricians, social scientists and an anthropologist, providing diverse perspectives in data collection and interpretation. Clinical researchers were mindful of the authority associated with their professional roles and sought to build trust with participants, while non-clinical researchers were often perceived as more impartial which helped facilitate open discussions on sensitive issues such as mistreatment by healthcare staff.

Reflexivity was approached as an ongoing and intentional process. We recognised that our assumptions were partly shaped by exposure to international models of birth companionship, particularly those emphasising continuous partner involvement, and we critically examined how these assumptions might influence our interpretation of the Malawian context. Regular team reflections and fieldwork debriefings allowed us to assess, question and refine emerging interpretations, especially when initial assumptions did not align with participants’ lived realities.

To strengthen analytical rigour, we used reflexive journaling, peer debriefing and triangulation across data sources. Consistent with reflexive thematic analysis, these strategies were not intended to remove researcher subjectivity, but to support critical engagement with how interpretations were shaped, enhancing the transparency, depth and grounding of the analysis. We adopted a co-constructive approach, recognising that researcher–participant interactions shaped both questions and responses.^38 39^

### Data collection and participants

These data form part of the ALERT study and were collected between December 2020 and January 2021 in maternity facilities selected for the wider study. The tools were originally informed by the WHO’s recommendations on intrapartum care for a positive childbirth experience and adapted to the Malawian context.^5^ They explored women’s childbirth experiences, provider interactions with labouring mothers and their companions, perceptions of care quality, respectful maternity care and the presence and role of companions during labour and birth. Observation guides captured ward conditions, clinical processes and interpersonal dynamics. All tools were pretested in comparable Malawian maternity settings and revised for clarity, relevance and consistency (details are provided in onlinesupplemental materials 1^4^). Interviews and focused group discussions (FGDs) were conducted in Chichewa, a widely spoken language in Malawi.^40^

Data were collected by a trained qualitative research team comprising two researchers with social science training and one experienced midwife (Who is the first author). All team members received training on the study objectives, interview guides, ethical conduct, informed consent, confidentiality and reflexive interviewing. The wider author team supported tool development, supervised fieldwork and engaged in regular reflexive debriefings to ensure quality and consistency. These debriefings were used to discuss emerging findings, field experiences, interviewer assumptions and how the researchers’ professional backgrounds and positionalities may have shaped interactions with participants and interpretation of the data.^41^

The dataset included interviews, FGDs, provider diaries and observations of maternity care providers, postnatal women and companions. Providers were purposively sampled to ensure variation in cadre, experience and work shifts, and included midwives, clinical officers, matrons and anaesthetists. Women were eligible if they had recently given birth at a study hospital to a baby weighing over 1000 g or above 28 weeks’ gestation. This paper focuses on women who had a companion during labour or birth, with women interviewed individually and companions participating in FGDs.

Data collection included semi-structured interviews with 14 maternity care providers and 24 postpartum women. Four FGDs were conducted, two with providers and two with birth companions at each facility, each lasting between 45 and 50 min. A few of the interviewed providers also participated in FGDs. To complement these insights, researchers gathered observational data over 72 hours by shadowing maternity care providers and accompanying labouring women. Furthermore, four maternity care providers in both facilities were invited to keep daily diaries on their care delivery experiences. After completing seven consecutive days of diary entries, these participants took part in follow-up FGDs to elaborate on themes that were developed from the initial data collected. Observations captured the experiences of birthing mothers, the environment, provider–patient interactions and routine care practices within the maternity wards. The extended observation period allowed participants to become accustomed to the researchers’ presence, helping to reduce changes in behaviour. While individual interviews provided in-depth personal accounts, FGDs encouraged shared and contrasting perspectives, and these were complemented by field observations that captured real-time behaviours and interactions in the maternity ward.

The sample size for this study was guided by the principle of information power as shown in table 1. This approach takes into account five dimensions, including the clarity of the study aim, the specificity of the sample, the quality of data obtained, the planned analysis and the relevance of the research context.^42^ These considerations informed our decisions throughout the study design, shaping a focused research aim, guiding purposeful sampling and ensuring that data collection and analysis aligned with the research aim.

**Table 1:** Overview of data collected

Participant group	Data collection method	Facility distribution	No. of participants	Counting note	Age range	Occupation/cadre
Mothers	Individual interviews	Government: 12; faith-based: 12	24	Unique interview participants	17–46	Teacher: 1; student: 1; farmer: 7; business owner: 5; housewife: 10
Birth companions	FGDs	Government: 10; faith-based: 10	20	Unique FGD participants	37–58	Business owner: 8; farmer: 11; housewife: 1
Maternity care providers	Individual interviews	Government: 7; faith-based: 7	14	Unique interview participants	24–39	Nursing officers, nurse–midwife technicians, clinical officers, anaesthetist
Maternity care providers	FGDs	Government: 7; faith-based: 7	14	Includes 7 providers who also participated in interviews; 7 were additional unique providers	24–39	Mixed maternity care provider cadres
Maternity care providers	7-day diaries and follow-up FGDs	Government: 3; faith-based: 3	6	Subset of provider participants; not added to total sample size	24–39	Mixed maternity care provider cadres
Total unique interview/FGD/diary participants	Interviews, FGDs, 7-day diaries	Both facilities	65	24 mothers+20 companions+21 unique providers	17–58	—
Mothers	Observation/go-along shadowing	Government: 8; faith-based: 8	16	Reported separately to avoid double-counting	17–46	Farmer, business owner, housewife
Maternity care providers	Observation/go-along shadowing	Government: 8; faith-based: 8	16	Reported separately to avoid double-counting	—	Nursing officers, nurse–midwife technicians, clinical officers, anaesthetist
Total observed participants/encounters	Observation/go-along shadowing	Both facilities	32	Observational component reported separately from interview/FGD/diary sample	—	—

FGD, focused group discussion.

### Data analysis

All interviews and focus group discussions were audio recorded, transcribed verbatim, translated into English by trained research assistants and anonymised before being uploaded into NVivo V.14 for coding. The first author led the analysis, with active involvement from other team members through iterative coding and regular reflexive discussions. These discussions allowed the team to compare interpretations, question assumptions and reflect on how researcher positionalities shaped the analytical process.

We used reflexive thematic analysis to understand how birth companionship was experienced, negotiated and given meaning. Analysis began with repeated reading of transcripts, provider diaries and observation notes to become familiar with the data. This was followed by open, data-driven coding to identify key ideas, emotions, tensions, experiences and meanings related to birth companionship. Themes were developed iteratively through the research team’s interpretive engagement with participants’ accounts, rather than by fitting the data into predefined categories.^43^ This initial process was flexible, and we developed new codes throughout the process. This was an important step to stay close to the data without imposing predefined or semi-predefined codes.

As coding progressed, the research team identified recurring differences in how companion participation was permitted, restricted, managed and negotiated within maternity care settings. These patterns suggested deeper power dynamics of inclusion and exclusion that could not be fully captured through descriptive themes alone. To deepen interpretation, we applied Gaventa’s Spaces of Power Framework as a guiding lens to explore how authority and voice are distributed across contexts. Rather than using it as a rigid coding structure, it served as a sensitising framework to support the analysis and interpretation of findings.^27^ Closed spaces refer to situations where providers or institutional routines control whether companions can enter, remain or participate in care. Invited spaces describe situations in which companions are allowed to support women, but only within roles and boundaries defined by providers or the facility. Contested spaces capture moments when companions seek visibility, ask for explanations, or attempt to influence care decisions, often generating tension within provider-controlled practices.

Unlike theories that focus narrowly on individual agency or institutional rules, Gaventa’s framework allowed us to connect everyday practices to broader power relations by categorising them into closed, invited and contested spaces. This illuminated how participation was shaped by those who created these spaces, who was allowed to enter and the level of influence companions could exercise. Use of this framework helped us to interpret how formal rules, institutional routines and provider discretion determined whether companions could speak, act and be heard.

Trustworthiness was supported using Lincoln and Guba’s criteria.^43^ Credibility was enhanced through multiple data sources, including interviews, FGDs, observations and provider diaries, and triangulation across women, companions and providers. Dependability was strengthened through detailed reporting of the study design, sampling, tool adaptation, data collection language, transcription, translation and stepwise analysis, enhancing transparency and traceability. Confirmability was strengthened through team reflections, field debriefs, reflexive journaling and shared analytical responsibility, helping to ensure that interpretations remained grounded in the data while acknowledging researcher reflexivity. Transferability was enhanced through rich contextual description of the study setting, including facility type, birth volumes, staffing patterns, shift systems, and the broader Malawian maternity care context.

## Results

The findings are presented across three overarching themes, which reflect varying degrees of inclusion, negotiation and tension in the roles of companions, shaped by structural and relational dynamics. Refer to table 2 for a summary of findings.

**Table 2:** Summary of findings

Theme	Subtheme	Codes
Companion inclusion is overlooked in maternity care design and policy, limiting continuous support.	Maternity ward design, policies and routines perpetuate a provider-centred model that limits the inclusion of companions.	Absence of formal guidelines on companion involvement, leaving it to provider discretion—rely on personal judgement. No formal companion training or orientation—untrained companions may hinder rather than help. No designated space at the hospital for companions making them feel excluded. Available but inadequate basic facilities—limited access to shelter, beds, restrooms and food often forces companions to incur additional costs. Restricted visiting hours disrupt continuous support for labouring women. Limited space in delivery wards—overcrowding prevents companion presence.
	Harsh, hierarchical provider communication silences companions and limits their participation.	Provider resistance to inclusion—lack effort to integrate companions into the care team. Providers view companions as disruptive—seen as obtrusive rather than helpful. Providers unwelcoming and rude to companions. Providers asking accusatory questions. Companions remaining silent when providers shout. Companions choosing not to raise concerns to protect the patient. Providers not listening to what the companion says. When companions speak up about the labouring mother, they are disciplined by providers.
Companion involvement in maternity care is permitted, yet carefully managed and limited by healthcare practices.	Permitted emotional and practical support across labour, recovery and newborn care within defined roles.	Offer emotional support (eg, reassurance, praise, comforting words). Provide non-pharmacological pain relief (eg, handholding, massage, breathing techniques), help create a calm and supportive environment. Encourage movement and position changes during labour. Advocate for the labouring woman’s wishes and preferences. Assist with hydration and comfort (eg, offering water, wet cloths, adjusting pillows). Bridge communication gaps between providers and labouring mothers. Facilitate connection with the woman’s partner and family. Escorting woman to hospital. Helping after surgery when woman cannot do things herself. Carrying the baby. Helping with baby care. Expressing breastmilk for baby and taking milk to the baby.
	Companions are invited only when their presence serves the practical needs of the ward.	Companions observe and offer support passively, with little guidance or direction from providers. Providers recognising companions only when convenient limiting proactive involvement. Companions seen as important for consent. Companions called only during crises to act as witnesses. Being used to calm or persuade the woman. Companions being treated as visitors rather than carers. Companions not treated as part of the whole process. Companion efforts to support women in distress are tolerated but go unrecognised or unappreciated.
Ambiguous companion roles lead to ongoing negotiation and tension over participation, authority and boundaries.	Role confusion and boundary overstepping by companions.	Companions occasionally take on clinical tasks during labour (eg, assist delivery), creating blurred professional boundaries and raising safety concerns among providers. Lack of defined roles leads to confusion and tension between providers and companions regarding appropriate responsibilities. In the absence of companion support, providers are pulled into non-clinical tasks, diverting attention from essential medical duties. Providers experience mixed feelings, sometimes viewing companions as helpful, other times as interfering or burdensome.
	Companions negotiate visibility, understanding and influence within provider-controlled care	Providers and companions struggle to understand each other. Companions mediate when women and providers are not agreeing. Companions wanting to witness what happens in labour. Companions asking why mode of birth is decided. Companions demanding transparency from providers. Companions requesting staff to reassess rather than just send women away. Companions acting as intermediary between provider and woman.

### Companion inclusion is overlooked in maternity care design and policy, limiting continuous support

Participants described a maternity care system in which ward design, policies and routines reinforced a provider-centred model that limited companion inclusion. Although companionship was recognised as valuable, it was not embedded within formal guidance or routine childbirth care. In the absence of written rules defining companions’ roles, both providers and companions operated in an unclear space where involvement depended largely on provider discretion, workload, staffing levels and personal judgement.

There’s no clear rule about what companions should do. Sometimes we involve them if we are short-staffed or if the situation allows, but most of the time it depends on the midwife on duty. We’re just using our judgment because there’s nothing written to guide us. (in-depth interview (IDI), maternity care provider)

Companions expressed often feeling unsure about what they were allowed to do when they entered the maternity ward. Many said they received no guidance or instructions and were uncertain if their presence was even welcome. They therefore tended to stay passive, even though they were willing to help. This lack of clarity made some feel like a burden and highlighted the unequal power dynamics, where healthcare providers made most of the decisions and controlled how things worked, leaving little room for companions to be involved.

When we arrived, no one explained what we were supposed to do. We had to figure things out by asking others, but without direction, it was hard to know how to help. (FGD, companion)

Companions and women described companionship as constrained even when companions were physically present, as labour management often prioritised provider workflow over continuous support. Companions were made to wait outside or in designated areas during assessments, ward transfers or before active labour admission, leaving women alone during periods of pain, fear or limited mobility. Their presence was also restricted during overcrowding or busy periods, while visiting hours further limited women’s access to continuous support during critical stages of labour.

I was in a lot of pain and could hardly move, but my companion was told to stay outside. At that time, I felt alone because I needed someone to help me and be near me most. (IDI, mother)

There’s no proper place for us, no space in the labour ward, no kitchen, no place to sleep. Some companions travel far, only to find overcrowded shelters. Sometimes we sleep outside, even in the rain. It’s hard to stay and support the women like we’re supposed to. (FGD, companions).

The physical environment was also reported to constrain companion participation. Facilities lacked adequate resting areas, toilets, shelter, food and water for companions. Companions often had to fend for themselves, relying on external resources for meals and resting in uncomfortable or unsanitary conditions. This placed additional financial and logistical burdens on them.

Toilet for companions were visibly unclean and lacked necessary supplies. Additionally, companions were observed leaving the facility to purchase food or water, as there were no on-site provisions or shops. (observation notes)

Harsh and hierarchical provider communication further shaped the conditions under which companions could participate. Rather than being actively welcomed into care, companions were sometimes perceived as disruptive, burdensome or difficult to manage. Providers described companions as needing supervision or ‘complicating’ care, particularly in overcrowded wards. Companions, in turn, felt dismissed or reprimanded when they asked questions or tried to speak on behalf of women. Many companions remained silent when reprimanded or avoided raising concerns to protect women from possible consequences. Even when they voiced legitimate concerns, they often felt ignored or disciplined.

Some companions just stand around or try to observe. They don’t know what to do, and sometimes they make things harder for us. If they become too much, we just ask them to wait outside …companions are difficult, unhelpful and really complicate the care we’re trying to provide. You end up doing double the work, attending to both the patient and the companion…The space is limited for them…midwives already know the best care to provide… It can be a real disruption. (IDI, maternity care provider)

We are just being taken as guardians who have come from the village to disturb the nurses…They treat us as if there is nothing fruitful that we can do, they dismiss us when we speak. (FGD, companions)

### Companion involvement in maternity care is permitted, yet carefully managed and limited by healthcare practices

Companions were permitted into maternity care and widely recognised as helpful, but their participation happened within clear boundaries set by the hospital and providers. As reflected in women’s, companions’ and providers’ narratives, companions were allowed to support women in ways that were seen as appropriate and useful, especially through emotional and practical care. Companions facilitated communication between the woman and the care team and assisted with basic comfort measures.

I came with my mother. She is the one who kept walking with me back and forth when they said it wasn’t time yet. She fetched me water to drink, encouraged me and provided updates to the people back home. (IDI, mother)

However, companion involvement usually occurred on providers’ terms. Support was welcomed when it aligned with clinical workflow or met immediate ward needs, but proactive involvement was often discouraged. This created a one-directional relationship in which companions were useful when called on but rarely treated as collaborators in care.

They chase you away for disturbing them but call you back when there’s a problem. (FGD, companion)

Participants described companions as mostly remaining on the sidelines, offering quiet support with little guidance about what they could or should do. Their involvement became visible mainly during specific moments, such as when consent was needed, when women required calming or persuasion or when providers wanted someone to witness a crisis. Outside these moments, companions were rarely recognised as part of the ongoing care process.

Most of the time I was just there watching and trying to help quietly, but no one really told me what I was supposed to do. They only called me when they needed something. (FGD, maternity care provider)

Sometimes companions are helpful, they calm the woman or help us communicate, but other times they get in the way or need constant watching. (IDI, maternity care provider)

Companions were positioned more like visitors than participants in care, even when they were actively supporting women through pain, fear and distress. Their contributions were tolerated but not always acknowledged or valued. Participants described a pattern in which companions could be present and useful, but only in limited and selective ways determined by providers and institutional routines.

We allow them to be around and support the woman, but their role is limited. Mostly, we involve them only when they are needed for a particular task or decision. (IDI, maternity care provider)

### Ambiguous companion roles lead to ongoing negotiation and tension over participation, authority and boundaries

Participants also described the complexity of shared caregiving spaces, where unclear role boundaries created tension between providers and companions over who could participate in childbirth care, how far that participation could go and who had the authority to decide. Without formal guidance, companions sometimes moved beyond emotional or practical support into tasks that providers considered clinical. In extreme cases, companions assisted during childbirth or engaged with aspects of labour monitoring when staff were unavailable. Companions reported being unprepared for the physical and emotional demands of these expanded responsibilities. These situations also contributed to provider stress, particularly when safety or protocol adherence was perceived to be at risk.

The woman who stayed next to me at the guardian shelter ended up helping her daughter give birth. They kept going to the labour ward but were turned away each time, and eventually, the baby was born in the corridor. With no nurses around, she had to deliver her own grandchild by herself. (FGD, companions)

At the same time, the absence of clear guidance also created problems in the opposite direction. When companions were absent or excluded, providers had to assume non-clinical support tasks such as helping women move, offering comfort, ensuring food and medicine were available, or assisting with newborn care, which diverted attention from their core clinical duties. Providers described feeling overwhelmed as they tried to balance these responsibilities with clinical duties. This contributed to emotional strain and sometimes burnout. Providers therefore expressed mixed views, seeing companions as both helpful and necessary, but also as interfering, burdensome or difficult to manage.

When there was a woman without a companion, I took it upon myself to make sure she got her medicine and food, and that her baby was looked after. But the ward was so busy, I couldn’t manage everything on my own, I ended up feeling completely overwhelmed. (FGD, maternity care provider)

These tensions became especially visible when companions tried to negotiate understanding and influence within provider-controlled care. Across participant groups, misunderstandings arose because providers and companions lacked shared expectations about the companion’s role. Companions often stepped in as intermediaries when women and providers disagreed, particularly when women were in pain, distressed or unable to speak for themselves. Mothers also described being affected by these tensions. When providers responded harshly to companions, women felt more anxious and less supported.

What made it harder for me was the tension around me… When my companion tried to speak and the providers responded harshly, I became more afraid and more stressed, because instead of feeling supported, I felt like I was becoming a problem. (IDI, mother)

There are times when companions are very helpful, especially when the woman is in too much pain to speak for herself. But their wanting to know what is happening can also be a distraction, especially when we are trying to manage urgent care. (IDI, maternity care provider)

Companions wanted to witness what happened during labour, understand clinical decisions such as caesarean section, and receive clearer explanations from staff. Some questioned why women were repeatedly sent away or asked providers to reassess them when labour pain intensified. These actions were not merely attempts to be present; they reflected efforts to gain visibility, understanding and some influence in care. Yet such efforts were often interpreted as interference.

I wanted to know why they were saying she needed an operation and why they kept sending her back, because she was in pain. But when you ask too much, it is like they think you are interfering, yet you are only trying to understand and help. (FGD, companions)

## Discussion

This study explored how childbirth companionship was enacted in facility-based maternity care, and how ward design, institutional routines, provider practices and everyday interactions shaped companions’ inclusion. Findings show that companionship was recognised as beneficial but was not fully integrated into routine care. Instead, companions entered maternity spaces selectively and inconsistently, with participation largely controlled by providers and constrained by unclear guidance, overcrowding, restrictive routines and hierarchical communication. Using Gaventa’s spaces of power framework, the findings are discussed to show how participation occurred through closed, invited and contested spaces, where access did not necessarily translate into meaningful influence.^27^

Viewed through Gaventa’s notion of closed spaces, companion inclusion was constrained not only by policy gaps but also by provider-controlled and facility-controlled decisions about who could enter, how long they could remain and what roles were considered legitimate. In the absence of clear national or institutional guidance, companion participation depended on individual providers’ discretion and was shaped by workload, staffing pressures and personal preference. Similar patterns have been reported in Burkina Faso and Kenya, where labour companionship was inconsistently implemented and influenced more by institutional culture than by women’s preferences.^20 44^ This policy vacuum reinforced hierarchical power relations, created uncertainty for both providers and companions and privileged clinical workflow over women’s need for continuous support. Formal role definitions, structured orientation and institutional backing may help integrate companions more effectively into woman-centred maternity care.^24 19 45^,^47^

Closed spaces were further reinforced by structural and infrastructural constraints. Overcrowded wards, restricted access to labour areas and the absence of basic amenities limited companions’ ability to remain present and provide sustained support. These conditions conflict with WHO guidance, which recommends a companion of choice throughout labour and childbirth as part of woman-centred intrapartum care.^48^ The findings are consistent with studies from Malawi and other low- and middle-income settings showing that lack of space, privacy concerns and inadequate facility preparedness undermine implementation of labour companionship, even where policies appear supportive.^4445 49^,^53^ This suggests that policy support alone is insufficient without practical investment in infrastructure, space, privacy, and logistical planning.

The findings also reveal hidden power within maternity care. Companion exclusion was shaped not only by routines and spatial constraints, but also by providers’ perceptions of companions as disruptive, burdensome or lacking legitimacy. Companions who sought clarification or spoke on behalf of women were often dismissed or reprimanded, reinforcing the view that silence was safer than participation. This reflects broader evidence that labour companionship is often constrained by provider concerns about disruption, role confusion and loss of control within medicalised models of childbirth that privilege biomedical authority over women’s emotional and social support needs.^944 54^,^56^ Thus, companion exclusion is not simply an interpersonal issue, but a manifestation of broader power relations embedded in maternity care.

Despite these exclusionary practices, companions also occupied partial invited spaces. They were permitted to support women and sometimes assist providers, but their participation remained informal, reactive and conditional. Without clear guidance or recognition, companions were often confined to passive roles, offering emotional and practical support only when allowed. This mirrors findings from Sweden and sub-Saharan Africa, where companions’ involvement fluctuated according to perceived usefulness rather than a shared model of care.^24 41 57^ Although continuous labour support is known to improve women’s childbirth experiences, the absence of structured integration left many companions feeling sidelined and undervalued.^58^ This reflects Gaventa’s concept of invited spaces, where participation is permitted but controlled by those in authority.

Unclear role boundaries also created contested spaces, where responsibilities shifted unpredictably and participation was negotiated in the moment between providers and companions. During staff shortages, some companions assumed tasks extending beyond emotional or practical support into areas closer to clinical care. While such involvement sometimes prevented women from being left unsupported, it also raised provider concerns about safety, accountability and adherence to protocol. Similar patterns have been described in resource-constrained settings, where overstretched services blur boundaries between lay support and professional responsibility.^9^ Without clearly defined roles, companionship can become both supportive and potentially problematic, creating tension rather than fostering collaboration. As a result, the responsibility for maintaining respectful, woman-centred care falls heavily on individual providers, increasing the risk of burnout and limiting meaningful collaboration between staff and companions.^5 50 55 56^

The findings highlight a paradox in how providers experienced companions in that they were viewed as both necessary and burdensome. When companions were absent, midwives often had to perform non-clinical tasks such as feeding, comforting and helping women with basic needs, increasing workload and emotional fatigue. Yet when companions were present, they could be perceived as disruptive, especially when they asked questions, sought explanations or challenged decisions. This reflects evidence from other low-resource settings, showing that providers may value companions as helpful supporters while also seeing them as additional responsibilities requiring supervision.^9 19 59 60^ These mixed perceptions were reinforced by the lack of clear policies and structured guidance, leaving providers to manage clinical and relational demands with limited institutional support.

The study further shows how hidden power and internalised hierarchies directly shaped women’s experiences. Many companions appeared to see themselves as tolerated rather than respected, remaining silent even in distressing situations, because speaking up risked dismissal or reprimand. This reflects broader sociocultural dynamics that position healthcare workers as unquestionable authorities and companions as passive bystanders.^6 19 61 62^ These strained interactions also affected mothers. When companions questioned delays or procedures and were dismissed, women reported feeling more anxious, unsupported or like a source of trouble. This aligns with respectful maternity care principles, which emphasise that positive childbirth experiences depend on respectful interactions among women, providers and support persons.^5^ The findings highlight the need for clear companion roles, institutional support and more respectful collaboration in maternity care.

Together, these findings highlight the need to shift maternity care from closed and selectively invited spaces toward systems where companion participation is recognised, structured and supported. This requires clear institutional policies defining companion roles, infrastructure that enables safe and continuous presence, and provider training that positions companions not as peripheral visitors, but as legitimate participants in maternity care.

### Strengths and limitations

A key strength of this study is the use of multiple qualitative methods, including IDIs, FGDs, observations and diaries, which enabled triangulation and provided a richer understanding of birth companionship from the perspectives of women, companions and providers. Following Braun and Clarke’s updated guidance on reflexive thematic analysis, this study embraced the interpretive nature of qualitative research, recognising that knowledge is co-produced between researcher and participant.^63^ The use of Gaventa’s Spaces of Power framework provides a clear analytical contribution that gives the analysis a strong conceptual lens for explaining how companionship is enabled, restricted and negotiated within maternity care, moving it beyond description to interpretation of power dynamics.

This study has several limitations. First, the data collection tool was originally developed for the broader ALERT implementation study and focused on responsiveness and professionalism rather than healthcare providers’ relationship with birth companions specifically. Although it generated relevant insights, it was not specifically designed to examine how power operates within birth companionship, which may have limited the depth of analysis on this issue. Second, women who laboured without companions, including those who may have been denied their preferred companion, were not a focus of this analysis.

## Conclusion

This study highlights that making birth companions a meaningful part of maternal care in Malawi requires more than policy change; it requires practical, multilevel action to overcome systemic and infrastructural barriers. Implementation is currently uneven because of limited resources, unclear guidance and institutional gatekeeping, producing environments where companions are tolerated but not truly integrated. This study demonstrates that companions operate within rigid, provider-driven systems that offer closed and tightly managed invited spaces, literally and figuratively. To meet WHO recommendations and embed companionship within woman-centred, respectful maternity care, health systems must redefine companion roles, invest in staff training and create physical and organisational infrastructure that enables shared power and trust.

## Supplementary material

10.1136/bmjph-2025-003706 online supplemental file 1

10.1136/bmjph-2025-003706 online supplemental file 2

10.1136/bmjph-2025-003706 online supplemental file 3

10.1136/bmjph-2025-003706 online supplemental file 4

## Data Availability

Data are available upon reasonable request.
