# Supplementary material for: Between help and hindrance: a qualitative study on inclusion of birth companions in closed, invited and contested spaces within maternity care settings in Malawi
Source: BMJ Public Health. 2026 Jul 10;4(3):e003706. doi: 10.1136/bmjph-2025-003706 (PMC13358271; doi:10.1136/bmjph-2025-003706)
Supplement: online supplemental file 1 [file bmjph-4-3-s001.pdf]

WP2

Overview of qualitative sub-studies – topics to be included and methodology

The generic tools for capturing user perspectives - women in childbirth and midwifes/nurses/doctors - in all four field countries should be seen as a starting point for country-specific contextualization. In this document we have listed the main topics to be addressed. During and after our kick-off discussions, we will elaborate the more specific questions to include. On demand, we can develop direct and indirect types of questions on the same topic, which will give country teams alternatives to pilot.

Part 1 Semi-structured interviews with mothers on childbirth experiences in a maternity ward

The topic guide is informed by WHO recommendations on intrapartum care for a positive childbirth experience (2018). Topics have been modified so they are presented in a neutral manner. We want to understand if the communication was effective between providers and women, but we do not mention the term effective. Another example is that emotional support from companions for women are deemed as globally important. We will investigate if/how emotional support is seen as important by the women. We will also ask about other kinds of support that women received/wanted from their companion such as advocacy and logistical support.

Table 1: Overview of topics and questions to women

Topics	Questions	Remarks
**Introduction to interview focus and process**	Place of childbirth, date, status of child<br>Background information on mother and child	Interview to understand women's' perspective<br>Questions on experiences of childbirth, support, challenges, modifiable behaviors<br>No right or wrong answers<br>Anonymous information<br>Interview about one hour by X who is a researcher
**Childbirth experience of mother**	Grand tour question on the childbirth – open ended: tell me about your childbirth from the beginning to the end?	Take note of the way the women describe the process (verbal and non-verbal clues, e.g. emotions) and which situations/transitions they emphasize. These should be followed up on

	Can be combined with specific open-ended questions: tell me about the first contact you had with a health staff upon arriving to the facility?	during the interview since they most likely capture experiences that were important for the women.
**Support from a companion**	Companion of choice? How decided who to go with you? Who else was considered?<br><br>What kind of support? Emotional, physical, logistical, communication/interaction?<br><br>What would you have preferred to be different? (the companion, support/lack of support, skill set of companion)<br><br>Barriers, willingness to change from companion, health staff? Enabling environment to allow companionship? How would the physical setting have needed to be improved to allow for that?	
**Communication with staff - responsiveness**	Language(s) spoken in facility with you?<br><br>Try to reconstruct how many health staff the woman has been in contact with:<br>Who was the first contact at the maternity ward?<br>What questions were you asked? How did you respond? What was difficult for you to talk about?<br>Did they call you by name? If yes, what name?<br>Do you recall if they were nurses/midwives/doctors?<br>Did staff come when you called them? How did they react to your calls?	

	How did they talk to you about any procedures done on you or your baby? How did they describe possible complications to you?<br><br>How were you encouraged during labor? During pushing?<br><br>Did providers talk about you in your presence? If yes, how did they talk about you? How did that make you feel?<br><br>Were staff disrespectful to you? In what ways? Tell me what happened?<br>Were staff complaining about something you did/did not?<br>Were you slapped? Yelled at?<br><br>Questions on specific situations of for communication:<br>- first contact when arriving to the facility<br>- first physical examination<br>- first bed<br>- childbirth<br>- childbirth final stage<br>- breastfeeding initiation<br>- transfer to maternity ward.<br><br>Among all the examples of communication with staff - what messages or actions made you the most comfortable?	
**Pain relief-options**	What pain relief options were offered, if any? Accepted? Why or why not? Costs?	

	Preferred compared to received? How talked about with who?	
**Perceived monitoring of labour including physical examinations**	How was the progress in labor monitored? What was done and by who? In what rooms/beds?<br><br>Did you experience complications during the childbirth? If yes, what were they? What was done to your knowledge? What was the outcome?	
**Fluid and food intake at the facility**	Timeline of enter/exit to facility?<br>Fluids/foods offered/brought with you/companion arranged?<br>Constraints/restrictions? Restrictions by whom?	
**Mobility and birth position of choice and instruction**	Position(s) of childbirth?<br>Who instructed? Alternatives offered? What would you have liked to try? Was the environment conducive for this position? Why?	
**Care of the newborn**	When the baby is born how did the staff refer to the baby?<br>When did you hold your newborn for the first time?<br>Did the newborn have any complications? What did you do? What happened?	
**Privacy**	How did you experience your privacy? At admission, during labor, at maternity ward?	
**Privacy**	How many providers have examined you before during or after childbirth? Do you recall names of	

	the providers? Do any standout as better/worse than others? If yes, in what ways?<br><br>Process of discharge: what was done/said/asked? Costs to pay?	
**Compare expectations to experience**	How would you compare your expectations of childbirth at this facility to your actual experience?<br><br>What did you hope for? What did you expect to happen although you hoped it wouldn't?<br>In general/on specifics.	
**Final questions to/from participant**	What have we not yet talked about? Please add.	

Methodology

Data collection method	Sampling principle (inclusion/exclusion)	Sample size[1]	Recruitment process	Data collector profile and numbers
**Semi-structured interviews with women who have delivered at an intervention maternity ward**<br><br>**Conduct 1-2 pilot interviews and revise accordingly.**	Purposive sampling based on the principle of maximum variation of types of childbirths/types of outcome (see below)<br><br>When there are more women available than what is needed for the study, in a second step, select women based on a simple	Select at least 3 women from each of the four predefined criteria. These are the minimum required and each country can add additional criteria based on interest and need.<br>= 12 women per hospital	Check the literature on respect of grieving period for women who experienced a loss. Recruit for interview in the postpartum room and get contact information to set up interview in the home.	Interviewer with social science and reproductive health background.<br>Only one person per interview is needed.<br><br>If interviewer only has limited experience with

[1] The concept of information power as coined by Kersti Malterud should be assessed in each country for each data collection. It is emphasized that sample size is a process to assess the quality of the data than a fixed number of participants.

	randomized procedure: for example on the day in the ward the first women who is eligible is approached, thereafter the second etc.<br><br>Consider including women who give birth on week-days/nights and week-ends.<br><br>Selection of women based on predefined inclusion criteria:<br>1. Vaginal uncomplicated childbirth according to staff<br>2. C-section<br>3. Fresh stillbirth, early newborn death<br>4. Complications during delivery but the baby survived including vacuum extraction or forceps delivery, delivery of premature baby	If it is assessed that there are major differences between the four selected hospitals in terms of HR profile of staff/ethnic differences or other aspects of the target population, the country team should consider to include 2 hospitals in the formative phase.	For women with complications and c-section they will stay at the hospital more than 24 hours and there will be more time to recruit.<br><br>Mobilization of women in the maternity, get contact information and set up appointment for interview in the home during the next week.	qualitative interviewing techniques it is recommended that two team members work together.
	Exclusion criteria:<br>Paying mothers if maternal care is provided free (MA)<br>Medical professionals working at the hospital			

	Additional inclusion criteria to be discussed:<br>Women with/without recommended ANC<br>Women who are first time to a hospital<br>First time mothers/third or more deliveries<br>Teenage mothers or women below 20 years at least if consent is an issue.			
