# Supplementary material for: Between help and hindrance: a qualitative study on inclusion of birth companions in closed, invited and contested spaces within maternity care settings in Malawi
Source: BMJ Public Health. 2026 Jul 10;4(3):e003706. doi: 10.1136/bmjph-2025-003706 (PMC13358271; doi:10.1136/bmjph-2025-003706)
Supplement: online supplemental file 2 [file bmjph-4-3-s002.pdf]

Appendix 1

ALERT: WP2- RESPONSIVENESS AND PROFESSIONALISM

RESEARCH QUESTION 2: How do nurse/midwives perceive the provided services during the childbirth process of care?

The main goal is to generate insights around childbirths within the maternity wards from the midwives' perspective. We propose to employ a variety of data collection methods to respond to this research question including semi-structured interviews with midwives, midwives journos (daily diaries)and focus group discussions to generate consensus and identify key domains emerging from the dairies. Details of the methods, sample size and recruitment processes are below:

Type of Study	Data collection method	Sampling Principle	Sample size	Recruitment Process	Data Collector Profile +Numbers
**Child birth at maternity: Perspectives of Midwives**	a) Semi structured interviews with midwives	**Purposive sampling based on the principle of maximum variation**<br><br>**Inclusion criteria:**<br><br>- Midwifes working in the maternity in sampled sites only<br>- Professionally trained midwives<br>- Male and female midwives<br>- Working on day and night shift<br><br>**Exclusion Criteria:**<br>- Not currently working in the maternity<br>- Maternity maids	**Segmented by:** We would like a variation in cadres. Adapt to your country context.<br><br>In MA it would include:<br>Gender =2 (I Male 1 Female)<br>Cadre=2 (1 Registered Midwife; Nurse Midwife Technician)<br>Placement =2 (1 in Labour-ward, 1 in postnatal ward)	1. Recruitment through Unit Matron & facility In charge.<br>2. Use staff rosters to select<br>3. Confirm interest to participate in the study<br>4. Confirm eligibility<br>5. Schedule time of interview after work shift or any other convenient time for the midwife	- 2 Data collectors/Facility<br>- Trained Nurse /Midwife + social scientist<br>- Data collector cannot be a Medical doctor

	b) Daily diaries with selected midwives (notes to be completed at the end of the day)	Inclusion:<br>-5 few selected midwives<br>- Not those previously interviewed	Select midwives a minimum of 1 facility= 5 midwives. For Malawi we will target CHAM and Public facility since the work dynamics are different (10 midwives).	Recruitment same as in 2 (a) above<br>- Midwives will be provided with a notebook and asked to reflect on their daily experiences by recording them in the note book	- Individual midwives will collect data through personal dairies
	c) Focus Group discussions to consolidate views and identify common domains emerging from the dairy notes (Optional)	**Inclusion criteria:**<br>- Those who participated in the dairy<br>**Exclusion:**<br>Those who did not participate in the dairy		- Midwives who participated in dairies	- Data will be collected by a Midwife with Qualitative skills.

PROPOSED OUTLINE OF TOPIC GUIDE

The topic guide is largely informed by Afulani et al.(2019) Person- Centered Maternity Care Scale (PCMC) including key responsiveness domains and the WHO recommendations on intrapartum care for a positive childbirth experience (2018). We propose to get midwives perspectives through vignettes type questions.

Table 1: Overview of topics and questions to midwives

Topics	Questions	Remarks
Introduction to interview focus and process	Place of childbirth, date, status of child<br>Background information on mother and child	Interview to understand women's' perspective

		Questions on quality of intrapartum care: Interactions, communication between midwives and mothers; Provision of physical, emotional and psychological support; Positive provider characteristics (empathy, motivation and competency); availability of essential physical resources and challenges.<br><br>No right or wrong answers<br><br>Anonymous information<br><br>Interview about one hour by X who is a researcher
Midwives experiences of child birth care	Open ended question on Midwives experiences with childbirth –tell me about your experiences with delivery of intrapartum care for pregnant women?<br><br>Specific questions can include their experiences with key stages of care:<br><br>- Admission of mothers during intrapartum care<br>- Processes of labour and delivery<br>- Postpartum care including initiation of breastfeeding	Take note of the way the midwife describes the processes of care, the barriers/enablers to care delivery.
Respectful and dignified care	- Open ended question: How do midwives feel about implementing RMC?<br>- Probe on Positive interactions between midwives and mothers/care givers. What are the influencing factors? How does this affect labour outcomes?	

	- Probe on experiences of negative interactions between mothers and midwives?<br>Eg. Mistreatment and abuse (verbal /physical)<br>- Explore occurrence of mistreatment and abuse<br>- Trigger factors?<br>- Variations in treatment of mothers by age, economic status, residence (rural /urban)<br>- Reporting structures in place for mistreatment and abuse?<br><br>Open ended question: In your opinion, what do you think about clients waiting time? Probe waiting time upon arrival to the facility/first physical examination/first bed/childbirth/breastfeeding initiation/transfer to postnatal ward)<br>- What strategies have been put in place to reduce waiting time?<br>- What is most challenging in implementing RMC? Why?	
Communication with mothers and their companions	-What are the midwives' experiences regarding communication to mothers and caregivers?<br>Probe: Language of communication<br>-How do midwives deal with mothers with language barriers? /Communication challenges?<br>-What are the midwives' experiences regarding provision of information to women during labour and delivery?<br>Hotspots for communication: first contact when arriving to the facility/first physical examination/first bed/childbirth/breastfeeding initiation/transfer to maternity ward)<br>- How is the information delivered?<br>- What strategies do midwives use to ensure that mothers and their caregivers have understood the information given?<br>- What challenges do midwives often face in communicating with mothers and guardians at the different hotspots? Explain	
Emotional/psychological/physical Support	What supportive care do midwives provide to women and care givers during childbirth?<br>Probe on:	

	- Emotional /psychological support eg. Information needs, allaying anxieties/fears<br>- Physical support if any?<br>(Other means of support)<br><br>What makes it easy for the midwife to provide supportive care to women during childbirth?<br>What circumstances make it difficult for the midwife to provide supportive care?	
Autonomy of care	What are the midwives' experiences regarding involvement of pregnant women in decision making around their care during childbirth?<br>Probe: What decisions would midwives likely involve mothers/caregivers?<br>- What makes it easy for midwives to involve pregnant women in decision making?<br>- What makes it difficult for midwives to involve pregnant women in decision making?<br>-What are midwives' experiences whether women are given opportunities to make choices?<br>- Choice of companion/choice of birth position, mobility in labour.	
Pain relief options	What pain relief options do midwives offer to mothers during labour, if any?<br><br>Accepted? Costs?<br><br>Preferred compared to received?	
Perceived monitoring of labour and newborn including physical examinations	What are the midwives' views regarding monitoring progress of labour? How is progress of labor monitored? What is done and by who?<br><br>Privacy/Confidentiality<br><br>-What are the midwives' views on securing privacy and confidentiality for mothers during intrapartum care?<br>- What is done to protect clients information?	

	- Whether permission/consent is sought for carrying out procedures/examinations on	
Care provision – degree of continuity	How do midwives ensure continuity of care?<br><br>What procedure is followed?<br><br>Process of discharge: what is done and by who? Timing of discharge from delivery room to postnatal ward.<br>How many providers a woman would need during their time in the hospital	
Social Accountability	What structures exist for mothers/caretakers to present their concerns at this facility?<br><br>What structures exist to provide oversight on issues of mistreatment and abuse?<br><br>Views on competencies of midwives in delivery of quality care?<br>Views on Staff motivation	
Final questions to/from participant	What have we not yet talked about? Please add.	
