# Supplementary material for: Between help and hindrance: a qualitative study on inclusion of birth companions in closed, invited and contested spaces within maternity care settings in Malawi
Source: BMJ Public Health. 2026 Jul 10;4(3):e003706. doi: 10.1136/bmjph-2025-003706 (PMC13358271; doi:10.1136/bmjph-2025-003706)
Supplement: online supplemental file 3 [file bmjph-4-3-s003.pdf]

Appendix 3

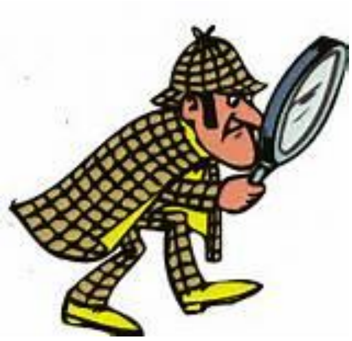

Shadowing clinicians in the maternity ward

Observations describe behavior, communication patterns, workflows and task of clinicians in the maternity ward. We can better understand individual and environmental constraints through observations. However, the observations provide only one piece of the understanding and needs to be complemented with interviews/group discussions to understand the rationale/justification of the observed behaviors.

<u>**Overview of topics and questions**</u>

Topics	Questions	Remarks
**Introduction to observations methodology to staff**	Understanding the organization of care to women<br>Collaboration between nurses, midwives, doctors	Issues of privacy and confidentiality<br>When we will observe and when not<br>Consent
**Exploratory observations without predefined behaviors/interactions to observe**		Field note taking. Use a notebook or phone to take descriptive notes. Add notes as soon as possible.<br><br>Use a column for reflective notes of the researcher where ideas are expressed.
**Examples of relevant observations:**		
	Understand workflow and interaction between cadres of staff at the maternity ward: Describe the tasks of different cadres.<br>When communicated and with whom and how (phone, direct, aspects of non-verbal and verbal communication, how avoid error? where are the disconnects, gaps, and opportunities of error?<br>Identify opportunities: what can be done better?	Identify area at maternity ward where communication between staff occurs and where documentation of workflow is done.
	Describe the clinical environment; different workstations of midwifes, nurses, doctors	Identify hot spots and re-distribute observers accordingly

	Draw map of the design of the maternity ward and the ways taken between stations by staff and patients;<br>Different sections of the maternity ward; who can see who?<br>Hotspot: whiteboard, morning/shift meetings	
	Describe interactions between clinicians and midwives? Or just staff in general and women:<br>Hotspots: admission; childbirth, breastfeeding initiation. Look at verbal and non-verbal clues, who is communicating with whom, how and when	
**Structured information on all midwives**	Age, gender, education, number of years of practice (of which at the x facility), languages spoken, number of children delivered	Offer staff to fill in on a paper version

<u>**Methodology**</u>

**Data collection method**	**Sampling principle (inclusion/exclusion)**	**Sample size[1]**	**Recruitment process**	**Data collector profile and numbers**
**Exploratory observations**	All clinicians are eligible to be included.<br><br>Spend full days at the maternity ward or as long time as possible. By spending several consecutive hours at the maternity ward the staff will get better used to the researcher and you will get access to an increasingly natural setting.	Number of clinicians to shadow should be continuously assessed according to the concept of information power.<br><br>Around 5 days (40 hours of observations) spent on the	Clinicians can be recruited at different times during their shift at the maternity ward.<br><br>Several staff can be shadowed during the same day – it will have to be tested but most	Fluent in the spoken language at the maternity ward (at least one of the spoken languages as applicable)

[1] The concept of information power as coined by Kersti Malterud should be assessed in each country for each data collection. It is emphasized that sample size is a process to assess the quality of the data than a fixed number of participants.

	Join during at least one night shift if possible.<br><br>Sampling principle is based on the characteristics of key informants:<br>○ Interest to participate<br>○ voluntary consent<br>○ able to express oneself<br>○ time<br>○ quality of rapport building with researcher	ward should be the first step; assess thereafter the rapport building and the quality of the data. Additional days to be added.	likely 3-4 staff per day.	

A few words on conducting observations:

<u>Types of researcher roles</u> are broad. Green (1958) conceptualized them in four overlapping ideal types (Gold 1958, in Green 2009: p.150)

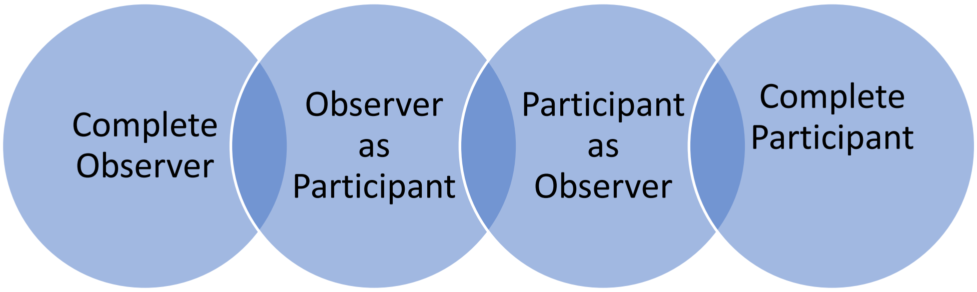

<u>How to organize an observation? (Lofland)</u>

- **Acts:** brief occurrences of action
- **Activities**: of longer duration
- **Meanings**: verbal accounts that participants use to define what is going on
- **Participation:** holistic involvement of participants in particular sets of acts and activities
- **Relationships**: Who is involved and with whom
- **Settings:** Descriptions of whole sites

What goes into fieldnotes?

- Observations of people’s doings and sayings
- Observations of people’s reactions to you
- Tape recordings, pictures, websites, artefacts, flyers, newspapers clippings, anything else that documents the cultural scene studied.
- Reflections on your own reactions

Different types of fieldnotes

- Condensed accounts (phrases, single words, unconnected sentences)
- Expanded accounts, filling details, recalling things not noted on spot. ASAP!)
- Fieldnote journal / diary, (personal side of fieldwork. Reflect on forms of presence)
- “Flying thoughts” (ideas, analysing and conceptualising. Place to think on paper)
