# Supplementary material for: Between help and hindrance: a qualitative study on inclusion of birth companions in closed, invited and contested spaces within maternity care settings in Malawi
Source: BMJ Public Health. 2026 Jul 10;4(3):e003706. doi: 10.1136/bmjph-2025-003706 (PMC13358271; doi:10.1136/bmjph-2025-003706)
Supplement: online supplemental file 4 [file bmjph-4-3-s004.pdf]

Appendix 2

Go-along women in the maternity ward

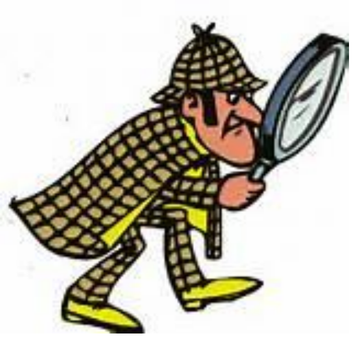

Observations describe behavior, communication patterns, and interactions between clinicians and women in the maternity ward. We can better understand individual and environmental constraints through observations. However, the observations provide only one piece of the understanding and needs to be complemented with interviews/group discussions to understand the rationale/justification of the observed behaviors.

<u>**Overview of topics and questions**</u>

Topics	Questions	Remarks
**Introduction to observations methodology to women**	Understanding the experience of care during childbirth	Issues of privacy and confidentiality<br>When we will observe and when not<br>Consent
**Exploratory observations without predefined behaviors/interactions to observe**		Field note taking. Use a notebook or phone to take descriptive notes. Add notes as soon as possible.<br><br>Use a column for reflective notes of the researcher where ideas are expressed.
**Examples of relevant observations:**		
	Describe interactions between women and clinicians and other staff at the maternity ward.<br>Describe interactions between woman and companion, if any.<br>Describe tasks that woman takes care of alone.	
	Describe the clinical environment; the personal/shared space of women<br>Draw map of the space assigned to woman; who can she contact and how?	

	Hotspots: admission; childbirth, breastfeeding initiation. Look at verbal and non-verbal clues, who is communicating with whom, how and when	
**Structured information on all women**	Age, sex, languages spoken, etc	See part 7
**List of follow-up questions**		Should we develop a guide on relevant follow up questions to women?

Methodology

Data collection method	Sampling principle (inclusion/exclusion)	Sample size[1]	Recruitment process	Data collector profile and numbers
**Participant observation of women from admission to discharge at the maternity ward**	All women are eligible to be included.<br><br>Spend full days at the maternity ward or as long time as possible. By spending several consecutive hours at the maternity ward the staff, women and companions will get better used to the researcher and you will get access to an increasingly natural setting.<br><br>The minimum hours are around 30 and can continue up to several months of observation and participation at the maternity ward. The longer period the more diverse data.<br><br>Sampling principle is based on the	Number of women to go-along (shadow) should be continuously assessed according to the concept of information power. The quality of the observations and the access to the interactions are essential.<br><br>Around 15-20 women would most likely be sufficient to observe patterns in the communication with staff.	Women can be recruited at different times during their stay at the maternity ward.<br><br>Several women can be shadowed during the same day – it will have to be tested but most likely 3-4 women per day.	Social scientist and knowledge of reproductive health and/or person with experience in making overt observations and ask questions along the process.<br><br>Fluent in at least one of the spoken languages

[1] The concept of information power as coined by Kersti Malterud should be assessed in each country for each data collection. It is emphasized that sample size is a process to assess the quality of the data than a fixed number of participants.

	characteristics of key informants:<br>○ Interest to participate<br>○ voluntary consent<br>○ able to express oneself<br>○ time			
**OR Maternity ward walk with recall questions of the experiences of the process of care with newly discharged mothers.**	If the go-along methodology is not accepted, an alternative approach is to ask women at discharge from the maternity ward to come back for a “maternity ward walk and interview” within a week to ten days. The maternity walk serves as a physical probe to trigger details and emotions during the different phases of the childbirth and stay at the maternity ward.			
